# HIF-1A and C/EBPs transcriptionally regulate adipogenic differentiation of bone marrow-derived MSCs in hypoxia

**DOI:** 10.1186/s13287-015-0014-4

**Published:** 2015-03-12

**Authors:** Chen Jiang, Jun Sun, Yafei Dai, Pengfei Cao, Liyang Zhang, Shuping Peng, Yanhong Zhou, Guiyuan Li, Jingqun Tang, Juanjuan Xiang

**Affiliations:** Cancer Research Institute, Key Laboratory of Carcinogenesis and Cancer Invasion of Ministry of Education, Key Laboratory of Carcinogenesis of Ministry of Health, Central South University, 110 Xiangya Road, Changsha, Hunan 410078 China; Hunan Cancer Hospital and the Affiliated Cancer Hospital of Xiangya School of Medicine, Central South University, Changsha, Hunan 410013 China; Hunan Key Laboratory of Nonresolving Inflammation and Cancer, Changsha, Hunan 410013 China; Department of Thoracic Surgery, the Second Xiangya Hospital of Central South University, Changsha, Hunan 410011 China

## Abstract

**Introduction:**

Bone marrow-derived mesenchymal stem cells (BMSCs, also known as bone marrow-derived mesenchymal stromal cells) are known to be a component of the tumor microenvironment. BMSCs are multipotent stromal cells that can differentiate into a variety of cell types, including osteocytes, chondrocytes, adipocytes, epithelial cells and endothelial cells. Stem cells found in niches or transplanted into injured tissues constantly encounter hypoxic stress. Areas with very low to no oxygen pressure exist in solid tumors. The differentiation capacity of BMSCs under hypoxic conditions remains controversial.

**Methods:**

In this study, a hypoxic workstation, set at an oxygen concentration of 0.2% was used to mimic the hypoxic microenvironment of cancer in vivo. Oil red O staining and alkaline phosphatase staining were used to examine the adipogenic or osteogenic differentiation, respectively, of BMSCs. Real-time PCR was performed to explore the expression of adipocyte- or osteocyte-specific genes. An RT2 Profiler™ PCR Array was used to screen a panel of 84 genes associated with human adipogenesis in BMSCs under normal and hypoxic conditions. A dual-luciferase reporter assay and chromatin immunoprecipitation (ChIP) were applied to analyze promoter activity to evaluate the possible regulatory mechanism of adipocyte-specific gene expression.

**Results:**

We found that this extreme hypoxia impaired osteogenic differentiation as indicated by the attenuation of alkaline phosphatase (ALP) activity and the reduced expression of osteogenic markers osteocalcin and osteopontin. Moreover, extreme hypoxia enhanced adipogenic differentiation, as indicated by the accumulation of lipid droplets and the expression of the adipocyte-specific genes leptin, LPL, CFD, PGAR and HIG2. In the extreme hypoxic conditions (0.2% oxygen), the overexpression of CCAAT enhancer-binding proteins (C/EBPs), especially C/EBPδ, and HIF-1A upregulated the promoter activities of adipocyte-specific genes such as leptin, CFD, HIG2, LPL, PGAR. In the present study, peroxisome proliferator-activated receptor-gamma (PPARγ) exerted a negative effect on the differentiation of BMSCs into adipocytes.

**Conclusions:**

In view of these findings, extreme hypoxia induced the adipogenic differentiation of BMSCs through HIF-1A and C/EBPs. These findings might provide clues regarding the roles of BMSCs in the cancer microenvironment.

## Introduction

At sea level the oxygen pressure is approximately 160 mmHg, whereas the oxygen pressure of tissues depends on the organ type. The oxygen pressure in normoxic tissue has been estimated to be 2 to 9% (14.4 to 64.8 mmHg) [1]. This normal tissue oxygen pressure can therefore be considered hypoxic from a molecular standpoint [2]. In some pathological conditions, such as heart disease, stroke, arthritis, wounds and tumors, oxygen deprivation is closely related to disease development. It has long been known that areas with very low or even zero oxygen pressure exist in solid tumors because aggressive tumor cells rapidly surpass the capacity of the nearest blood vessel. Tumor hypoxia appears to be strongly associated with tumor propagation, malignant progression and therapy resistance. Meanwhile, cancer cells have developed remarkable adaptive mechanisms to survive the severe hypoxia, including angiogenesis, autophagy and glycolysis.

Bone marrow-derived mesenchymal stem cells (BMSCs, also known as bone marrow-derived mesenchymal stromal cells) are known to be a component of the tumor microenvironment. Mesenchymal stem cells (MSCs) are multipotent stromal cells that can differentiate into a variety of cell types, including osteocytes, chondrocytes, adipocytes, epithelial cells and endothelial cells. Bone marrow-derived cells are crucial for the generation of a suitable microenvironment in the primary tumor, as well as for the development of metastasis [3-5]. Many factors participate in the regulation of MSC differentiation. Differentiated MSCs also regulate the biological characteristics of cancer cells, and adipose MSCs have the ability to differentiate into mature adipocytes and initiate cytokine signaling within the tumor microenvironment [6].

Hypoxia is an important microenvironmental factor in the fate of MSCs. The roles of hypoxia in the differentiation of MSCs remain controversial. However, to investigate the roles of MSCs in the tumor microenvironment, the effect of extreme hypoxia on the differentiation of MSCs must be elucidated. In this study, we set the oxygen pressure at 0.2% to study the differentiation of MSCs in this nearly extreme hypoxic environment.

## Methods

### Mesenchymal stem cell isolation and culture

Human BMSCs were obtained from bone marrow aspirates of ribs from patients undergoing thoracic surgery. The isolation and culture of MSCs were performed using methods described previously [7]. Samples were from the Second Xiangya Hospital, Central South University, Hunan, China. The patients were informed about the sample collection and signed informed consent forms. Collections and use of tissue samples were approved by the ethical review committees of Second Xiangya Hospital. BMSCs are a monolayer cultured in low-glucose Dulbecco’s modified Eagle’s medium (GE Healthcare Hyclone, Logan, Utah, USA), supplemented with 10% fetal bovine serum (Gibco, Life Technology, Shanghai, China), penicillin (100 U/ml) and streptomycin (100 mg/ml). Cells are cultured in 37°C in a humidified atmosphere of 5% carbon dioxide and are subcultured using 0.25% (w/v) trypsin–ethylenediamine tetraacetic acid solution. Osteogenic and adipogenic differentiation were performed using the differentiation media (Cyagen Bioscience Inc., Guangzhou, China). For hypoxia induction, BMSCs were incubated in 0.2% oxygen concentration at 37°C temperature, 5% carbon dioxide concentration and 90% humidity in a Hypoxic Workstation (Don Whitley, West Yorkshire, UK). Cells were lysed for extraction of protein and RNA in the workstation to avoid reoxygenation.

### Plasmids

Human CCAAT enhancer-binding protein (C/EBP) delta expression plasmid, which contains the full-length coding region of human C/EBPδ, was purchased from Origene (Rockville, MD, USA). Human hypoxia-inducible factor 1 alpha (HIF-1A) expression plasmid was purchased from Addgene (Cambridge, MA, USA). C/EBPα and C/EBPβ were constructed by inserting full-length coding regions into PCDNA3.1. The firefly luciferase reporter plasmid pGL3-Basic plasmid was purchased from Promega (Madison, WI, USA). For screening promoter activity, five reporter gene vectors with promoter segments were constructed. The putative promoters, about 1,000 base pair upstream fragments of genes such as leptin, CFD, LPL, HIG2 and PGAR, were amplified by PCR from genomic DNA that was isolated using the Genomic DNA Extraction Kit (Takara, Dalian, China). Primer sequences are presented in Table 1. Cloning of different fragments to pGL3-Basic (Promega), which is a vector carrying the firefly luciferase gene, was performed after the restrictive enzyme cut. Finally, all plasmids were confirmed by sequencing. For the HIF-1A short hairpin RNA construct, oligo (5′-CTGATGACCAGCAACTTGA-3′) was designed to construct the lentivirus gene transfer vectors. The double-stranded short hairpin RNA oligo was cloned into the BLOCK-iT™ lentivirus vector (Invitrogen, Carlsbad, CA, USA). The lentivirus vector was cotransfected with the lentivirus package plasmids into 293FT cells. Forty-eight hours post transfection, the virus containing supernatant was harvested by collecting the medium. For infection of BMSCs, the cells were cultured in six-well plates, and when the culture reached 80% confluence the concentrated lentivirus was added to the culture dishes.

**Table 1:** **PCR primers used for luciferase constructs**

	**Forward**	**Reverse**	**Amplified DNA fragment length (base pairs)**
LPL	5′-TACTCGAGATGTGCATGCCTCTTA-3′	5′-ATAAGCTTCAGGGCTTTGCTCTCC-3′	1,179
CFD	5′-TCCTCGAGTGACTCTGTTCATCAGAAC-3′	5′-GTAAGCTTCTACACAGCCCTGTCCCTC-3′	989
HIG2	5′-TCCTCGAGTCTTTAGTTC AAGCCG-3′	5′-ATAAGCTTCCGGAGGAAA GTCGGT-3′	1,008
PGAR	5′-GACTCGAGAAAGTCTCTCCTGGTC-3′	5′-ACAAGCTTGTTCCAGGTGCGAGGA-3′	969
C/EBPδ	5′-TGCTCGAGATCTGCTCTGCTTT-3′	5′-TCAAGCTTTGGAGTCGATGTAGGCG-3′	1,001
Leptin	5′-TACTCGAGATCATGTAAA GCGGGG-3′	5′-GCAAGCTTCAAGAAAGACCAGAGA-3′	918

### Inhibitors and chemicals

The peroxisome proliferator activated receptor gamma (PPARγ) inhibitor GW9662 and C/EBP inhibitor betulinic acid were purchased from Sigma (St. Louis, MO, USA).

### Flow cytometry

Flow cytometry was performed on BMSCs that were stained for CD44, CD105, CD34, CD45 and CD11b. The following antibodies specific for human molecules were used: PC5-CD11b (Beckman Coulter, Brea, CA, USA), FITC-CD44 (Beckman Coulter), PC7-CD45 (Beckman Coulter, CA, USA), ECD-CD34 (Beckman Coulter, Brea, CA, USA) and PE-CD105 (eBioscience, CA, USA). Flow cytometry was performed on the Moflo XDP (Beckman Coulter, Brea, CA, USA). The corresponding isotype control monoclonal antibodies were from Beckman Coulter.

### Induction of adipogenic and osteogenic differentiation

After induction with differentiation media or hypoxia treatment, BMSCs were fixed in phosphate-buffered saline containing 4% Paraformaldehyde and stained with Oil Red O (cyagen, Guangzhou, China) or Alizarin red (cyagen, Guangzhou, China). Alkaline phosphatase (ALP) activity was determined using an ALP Staining Kit (beyotime, Shanghai, China).

### Quantitative PCR and RT^2^ profiler arrays

Total RNA was extracted from cells and samples using Trizol® reagent (Invitrogen, Carlsbad, CA, USA). Real-time PCR was performed using the Bio-Rad IQ™5 Multicolor Real-Time PCR detection System (Bio-Rad, Shanghai, China). The data were analyzed using iQ5 software (Bio-Rad, Shanghai, China). The relative gene expression was quantified on the basis of the threshold cycle value and normalized using the housekeeping gene β-actin. Data are representative of the means of three experiments. Student’s *t* test was applied to compare two or more values; *P* <0.05 indicated that there was a significant difference. The quantitative PCR protocol was 95°C for 30 seconds and 40 cycles of 95°C for 5 seconds and 60°C for 30 seconds. A final melting curve analysis (60 to 95°C) was conducted. The standard curve was produced using a slope of approximately –3.32 (~100% efficiency). Primer sequences are presented in Table 2. The human adipogenesis RT^2^ Profiler PCR Arrays (Sabioscience, Shanghai, China) were used to profile the expression of 84 genes related to adipogenesis. RT^2^ Profiler PCR Arrays were performed to quantify mRNAs of multiple genes, including housekeeping genes, according to the manufacturer’s protocol. Total RNA was extracted from BMSCs in normoxic and hypoxic conditions with TRIzol. Single-strand cDNA was synthesized from 2 μg total RNA using the RT^2^ first-strand cDNA synthesis kit (Sabioscience, Shanghai, China). The cDNAs were mixed with RT^2^ Real Time SYBR Green/ROX PCR master mix (Sabioscience, Shanghai, China) and real-time PCR was performed in accordance with the manufacturer’s instructions. Thermal cycling and fluorescence detection were performed using the Bio-Rad IQ™5 Multicolor Real-Time PCR detection System, and expressions of adipogenesis-regulated transcripts were compared between the groups. Gene expressions with absolute fold change >2 were taken as differentially expressed genes between groups. The RT-PCR array data have been deposited in the public repository Gene Expression Omnibus [GEO:GSE65842]. The interactive network during the adipogenesis was constructed by Gene Network Central Pro™ (Sabioscience, Shanghai, China).

**Table 2:** **PCR primers used for gene expression**

	**Forward**	**Reverse**
OPN	5′-TTGCAGTGATTTGCTTTTGC-3′	5′-GCCACAGCATCTGGGTATTT-3′
OCN	5′-GACTGTGACGAGTTGGCTGA-3′	5′-CTGGAGAGGAGCAGAACTGG-3′
ALP	5′-CCACGTCTTCACATTTGGTG-3′	5′-GCAGTGAAGGGCTTCTTGTC-3′
PGAR	5′-TGCAAGATGACCTCAGATGG-3′	5′-CCATGATGCTATGCACCTTC-3′
HIG2	5′-CCACAGTGCAAGACTCCATC-3′	5′-GCCATACTGCTGAGGAAAGC-3′
PPARγ	5′-GAGCCCAAGTTTGAGTTTGC-3′	5′-CTGTGAGGACTCAGGGTGGT-3′
LPL	5′-AGTGGCCAAATAGCACATCC-3′	5′-CCGAAAGATCCAGAATTCCA-3′
β-actin	5′-ACTGGAACGGTGAAGGTGAC-3′	5′-AGAGAAGTGGGGTGGCTTTT-3′

### Western blot

The protein used for western blotting was extracted using RIPA lysis buffer (Beyotime Biotechnology, Shanghai, China) supplemented with protease inhibitors (Roche, Guangzhou, China). The proteins were quantified using the BCA™ Protein Assay Kit (Pierce, Appleton, WI, USA). The western blot system was established using a Bio-Rad Bis-Tris Gel system according to the manufacturer’s instructions. Rabbit-anti-human Hif-1A antibody was purchased from Santa Cruz (Santa Cruz, CA, USA); rabbit-anti-human Hif-1A antibody for chromatin immunoprecipitation was purchased from Abcam (Shanghai, China). β-actin antibody was purchased from Sigma. Primary antibodies were prepared in 5% blocking buffer at a dilution of 1:1,000. Primary antibody was incubated with the membrane at 4°C overnight, followed by wash and incubation with secondary antibody marked by horseradish peroxidase for 1 hour at room temperature. After rinsing, the Polyvinylidene Difluoride (PVDF) membrane carried blots and antibodies were transferred into the Bio-Rad ChemiDoc™ XRS system, and then 200 μl Immobilon Western Chemiluminescent HRP Substrate (Millipore, MA, USA) was added to cover the membrane surface. The signals were captured and the intensity of the bands was quantified using Image Lab™ Software (Bio-Rad, Shanghai, China).

### Luciferase activity assay

The firefly luciferase is widely used as a reporter of promoter activities by cloning interested promoters to the upstream of the firefly luciferase coding gene. The activity of experimental reporter (firefly) is normalized by the activity of the internal control (renilla). HEK293 cells were transfected with constructed reporter vectors simultaneously with plasmid vector (Promega, Madison, WI, USA) containing cDNA coding renilla luciferase, which is driven by CMV promoter. Forty-eight hours after transfection, cells were harvested with passive Lysis buffer supplied by the Dual-Luciferase Reporter (DLR™) Assay System (Promega), referring to the manufacturer’s instructions. An appropriate volume of cell lysate was added into a well of the 96 MicroWell™ Plates (NUNC, Roskilde, Denmark), followed by 25 μl LARII. The firefly luciferase activities were measured with a luminometer (TECAN, Männedorf, Swiss). The renilla luciferase activities were also measured. The relative luciferase activity was represented by the ratio of firefly luciferase activity to renilla luciferase activity. Each experimental group included three repeats and data are shown as means.

### Chromatin precipitation

The chromatin immunoprecipitation assay was performed using the Chromatin Immunoprecipitation Assay kit (EZ ChIP; Millipore) according to the manufacturer’s instructions. Briefly, HEK293 cells were transfected with pCDNA3.1-HIF-1A or human C/EBPδ. C/EBPδ plasmid construct was engineered to express the complete open reading frame with an expression Flag tag. Forty-eight hours after transfection, the cells were then cross-linked by 1% formaldehyde for 10 minutes. The formaldehyde was quenched using 2 M glycine for 5 minutes at room temperature before harvest. Cells were collected by centrifugation in phosphate-buffered saline containing protease inhibitors and were lysed in SDS-lysis buffer. Soluble chromatin was prepared after sonication to an average DNA length of 200 to 500 base pairs. Fragmented chromatin was immunoprecipitated using antibodies against HIF-1α (Millipore) or Flag together with Protein A/G PLUS-Agarose overnight at 4°C on a rotating platform. The agarose beads were washed, chromatin extracted and protein–DNA cross-links reversed. DNA was purified and was analyzed by PCR using the specific primers. Normal immunoglobulin G was used as a negative control. Anti-RNA Polymerase II was used as positive control. The total input was the supernatant from the no-antibody control.

### Statistical analysis

Statistical analysis was performed using the Statistical Package for Social Science-10 software (SPSS, Chicago, IL, USA) and GraphPad (San Diego, CA, USA). Data are presented as mean ± standard deviation of the results from three independent experiments. Results of gene expressions were analyzed by two-tail Student’s *t* tests. *P* <0.05 was considered statistically significant.

## Results

### Mesenchymal stem cells under hypoxic conditions showed the potential to differentiate into adipocytes

**Figure 1:**
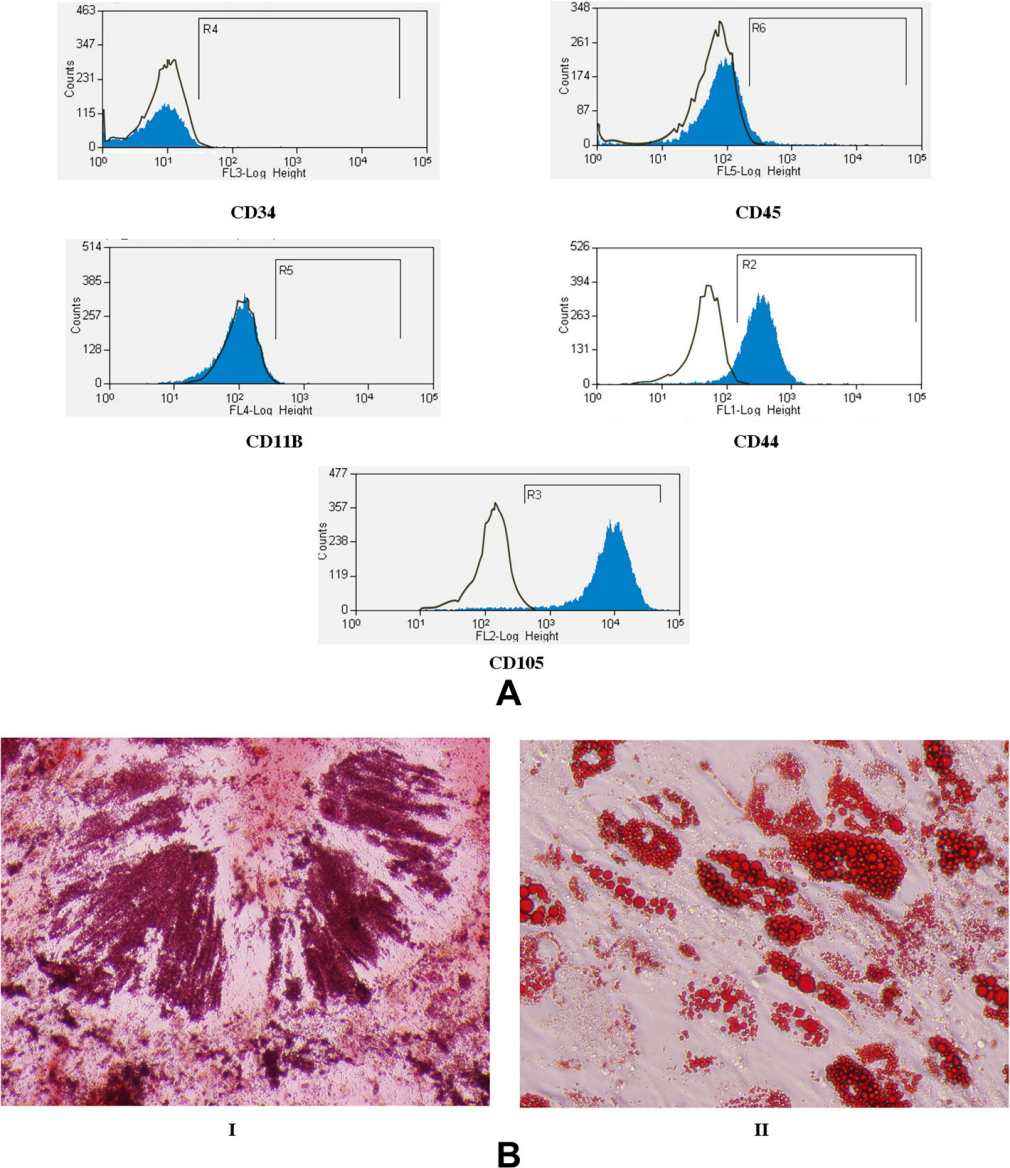
**Phenotypic analysis of bone marrow-derived mesenchymal stem cells. (A)** Bone marrow-derived mesenchymal stem cells (BMSCs) were analyzed by flow cytometry analysis for the surface expression of CD34, CD44, CD45, CD105 and CD11B. **(B)** BMSCs were tested for their differentiation potential into osteocytes and adipocytes when cultured in differentiation media. Alizarin red staining and Oil red O staining were performed. (I) Alizarin red S staining showed calcium deposition (red). (II) Oil red O staining revealed lipid droplets (red) in adipocyte-differentiated mesenchymal stem cells. Magnification: 200 ×.

BMSCs isolated from the ribs of patients undergoing thoracic surgery or bone marrow aspiration were screened by surface marker expression using flow cytometry. Immunophenotyping confirmed that the BMSCs displayed mesenchymal markers such as CD44 and CD105 and did not display the hematopoietic progenitor markers CD34, CD45 and CD11b (Figure 1A). The capability of these cells to differentiate into adipogenic and osteogenic lineages *in vitro* is displayed in Figure 1B. Osteogenic or adipogenic media induced the osteocalcin production or lipid droplet accumulation, respectively.

**Figure 2:**
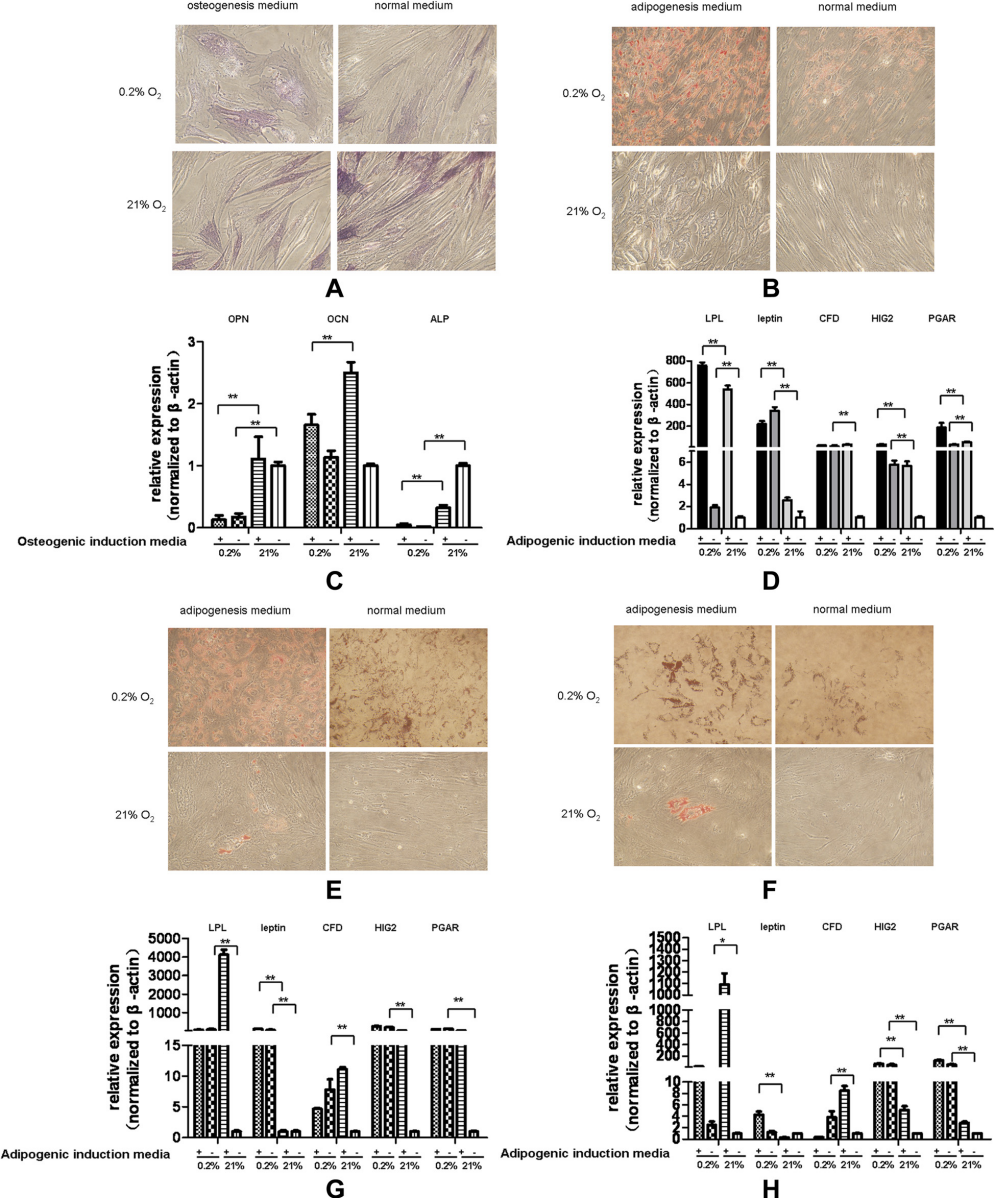
**Bone marrow-derived mesenchymal stem cells show potential to differentiate into adipocytes. (A)** Alkaline phosphatase staining showed less blue-stained osteocytes in bone marrow-derived mesenchymal stem cells (BMSCs) treated under hypoxia (magnification: 100×). **(B)** Oil red O staining revealed that the lipid accumulation in BMSCs was significantly higher under hypoxic conditions than normoxic conditions (magnification: 100×). **(C)** Real-time PCR assays showed that hypoxia decreased the mRNA expression of the osteoblast marker genes *ALP*, *OPN* and *osteocalcin*. Cells were treated in a hypoxic chamber for 3 days. **(D)** Real-time PCR results showed increased mRNA expression of the adipocyte-associated genes *leptin*, *LPL*, *CFD*, *HIG2* and *PGAR*. Cells were treated in a hypoxic chamber for 3 days. **(E)** Seven days after treatment, Oil red O staining revealed that the lipid accumulation in BMSCs was significantly higher under hypoxic conditions than normoxic conditions. **(F)** Fourteen days after treatment, Oil red O staining revealed that the lipid accumulation in BMSCs was significantly higher under hypoxic conditions than normoxic conditions. **(G)** BMSCs showed characteristics of mature adipocytes after long-term hypoxic treatment. Real-time PCR results showed increased mRNA expression of *leptin*, *HIG2* and *PGAR* after 7 days of hypoxic treatment. **(H)** Real-time PCR results showed increased mRNA expression of *leptin*, *HIG2* and *PGAR* after 14 days of hypoxic treatment. In contrast, the early markers of adipocytes such as LPL were expressed at lower levels in BMSCs under hypoxia compared with that of BMSCs treated with adipogenic differentiation media under normoxia after long-term hypoxic treatment. *p <0.05; **p <0.01.

To evaluate the effects of low oxygen levels, BMSCs were cultured under normoxia (21% oxygen) or hypoxia (0.2% oxygen). Three days after hypoxic treatment, ALP staining, Oil red O staining and real-time RT-PCR were used to assess the effects of hypoxia on osteogenic and adipogenic differentiation in BMSCs. BMSCs under hypoxic conditions showed less intense ALP staining and more intense Oil red O staining in normal media or differentiation media (Figure 2A,B). Oil red O staining revealed that the lipid accumulation in BMSCs was significantly higher under hypoxic conditions than normoxic conditions, even with short-term hypoxic treatment (Figure 2B).

As shown in Figure 2C,D, hypoxia decreased mRNA expression of osteoblast marker genes *ALP*, *OPN* and *osteocalcin* and increased mRNA expression of adipocyte-associated genes such as *LPL*, *leptin*, *HIG2*, *CFD* and *PGAR*. The results showed that extreme hypoxia enhanced the adipogenic differentiation of BMSCs and inhibited the osteogenic differentiation of BMSCs. We also performed Oil red O staining and real-time PCR to evaluate the adipogenic differentiation of BMSCs under hypoxic conditions for an additional period of 7 and 14 days (Figure 2E,F,G,H). BMSCs under hypoxic conditions showed more lipid accumulation than BMSCs under normoxic conditions. Treatment with complete media did not result in lipid accumulation under normoxia. After long-term hypoxic treatment, BMSCs expressed mature adipocyte markers such as leptin, HIG2 and PGAR. We noticed that when treated with adipogenic differentiation media, BMSCs under normoxia expressed more LPL compared with those under long-term hypoxia. This suggested that the early adipocyte marker LPL acts on preadipocytes during the early stage of BMSC differentiation. Hypoxia promoted the differentiation of BMSCs, and these BMSCs showed characteristics of mature adipocytes.

### HIF-1A transcriptionally regulated the adipogenic differentiation of BMSCs

**Figure 3:**
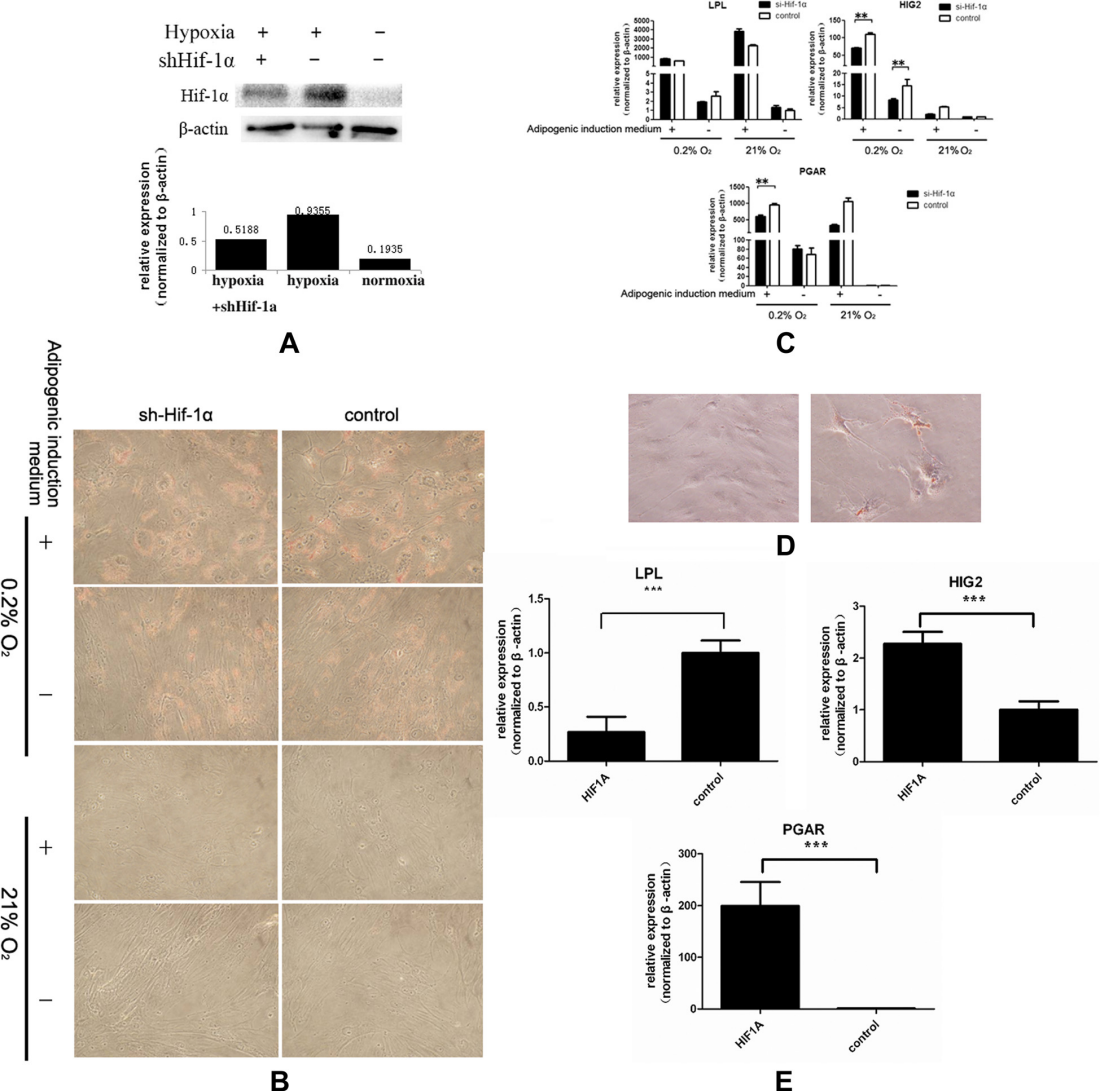
**HIF-1A regulates the adipogenic differentiation of bone marrow-derived mesenchymal stem cells. (A)** Stable hypoxia-inducible factor 1 alpha (HIF-1A) suppression in bone marrow-derived mesenchymal stem cells (BMSCs) was established using lentivirus-based delivery. **(B)** Oil red O staining revealed that BMSCs with knockdown of HIF-1A under hypoxic conditions showed less formation of lipid oil (magnification: 100×). **(C)** Real-time PCR results showed that hypoxia in the HIF-1A knockdown BMSCs induced decreased expression of HIG2 and PGAR compared with BMSCs expressing wild-type HIF-1A. **(D)** Oil red O staining showed intracellular lipid droplet accumulation after electroporation of a *HIF-1A* construct (magnification: 100×). **(E)** Real-time PCR assays showed that electroporation-mediated *HIF-1A* overexpression in mesenchymal stem cells induced the expression of *HIG2* and *PGAR*, but not *LPL*. **p <0.01; ***p <0.001.

HIF-1A plays an important role in the adaptive response of cells to hypoxia. We then investigated the effect of HIF-1A on the adipogenesis of BMSCs under hypoxic conditions. Stable HIF-1A suppression in BMSCs was established using lentivirus-based delivery (Figure 3A). HIF-1A knockdown BMSCs under hypoxic conditions showed less lipid droplet accumulation, indicating that HIF-1A may be involved in BMSC adipogenesis under hypoxic conditions (Figure 3B). In the HIF-1A knockdown BMSCs, hypoxia induced decreased HIG2 and PGAR expression compared with BMSCs expressing wild-type HIF-1A (Figure 3C). Electroporation-mediated HIF-1A overexpression in BMSCs induced lipid droplet accumulation (Figure 3D,E).

### Gene expression profiling of BMSCs under hypoxic conditions

**Figure 4:**
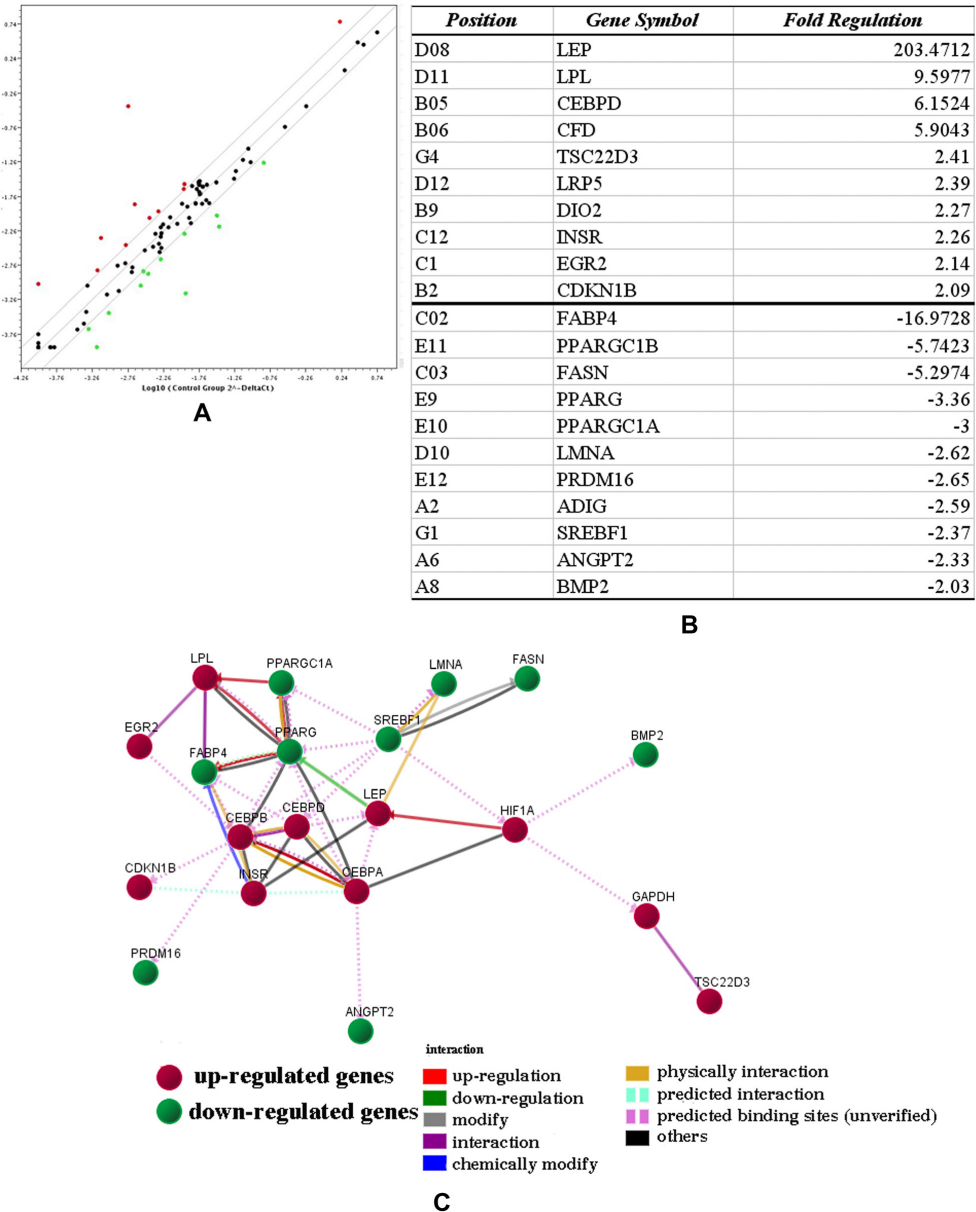
**Gene expression profiling of bone marrow-derived mesenchymal stem cells under hypoxic conditions.** An RT^2^ Profiler™ PCR Array (Sabioscience, Shanghai, China) was used to screen a panel of 84 genes associated with human adipogenesis in bone marrow-derived mesenchymal stem cells (BMSCs) under normal and hypoxic conditions. **(A)** Scatter plot of upregulation and downregulation of all of the investigated genes. Black line, fold change [(2^^^(^∆∆^Ct)]. **(B)** Ten genes were upregulated and 11 genes were downregulated by twofold in BMSCs under 0.2% oxygen compared with those under 21% oxygen. **(C)** The interactive network during adipogenesis.

To identify proteins involved in the regulation of adipogenic differentiation under hypoxic conditions, an RT^2^ Profiler PCR Array was used to screen a panel of 84 genes associated with human adipogenesis in BMSCs under normal and hypoxic conditions. Fold changes in gene expression were calculated using the ^ΔΔ^Ct method. The RT-PCR array data have been deposited in the public repository Gene Expression Omnibus [GEO:GSE65842]. As a result, 10 genes were upregulated, and 11 genes were downregulated by twofold in BMSCs under 0.2% oxygen compared with those under 21% oxygen (Figure 4A). Expression of the genes encoding leptin, LPL and CFD was dramatically increased under 0.2% oxygen. Expression of the genes encoding PRDM16 and BMP2 was decreased under 0.2% oxygen (Figure 4B). The mRNA levels of the adipogenic transcription factors C/EBPs, such as C/EBPα (fold change 1.87), C/EBPβ (fold change 1.79) and C/EBPδ (fold change 6.15), were significantly higher in BMSCs under hypoxic conditions compared with cells under normoxic conditions. However, the mRNA levels of PPARγ were decreased under hypoxic conditions (downregulated by 3.36-fold; Figure 4B). The interactive network during the adipogenesis is shown in Figure 4C. As mentioned, extreme hypoxia induces a proadipogenic effect. Adipogenesis is driven by a complex transcriptional cascade that involves the activation of C/EBPs, which are rapidly expressed after hypoxic treatment. However, PPARγ – the master adipogenic transcriptional regulator – may inhibit the differentiation of BMSCs into adipocytes under extreme hypoxic conditions.

### Adipogenic differentiation of MSCs is regulated by a C/EBP-mediated pathway

**Figure 5:**
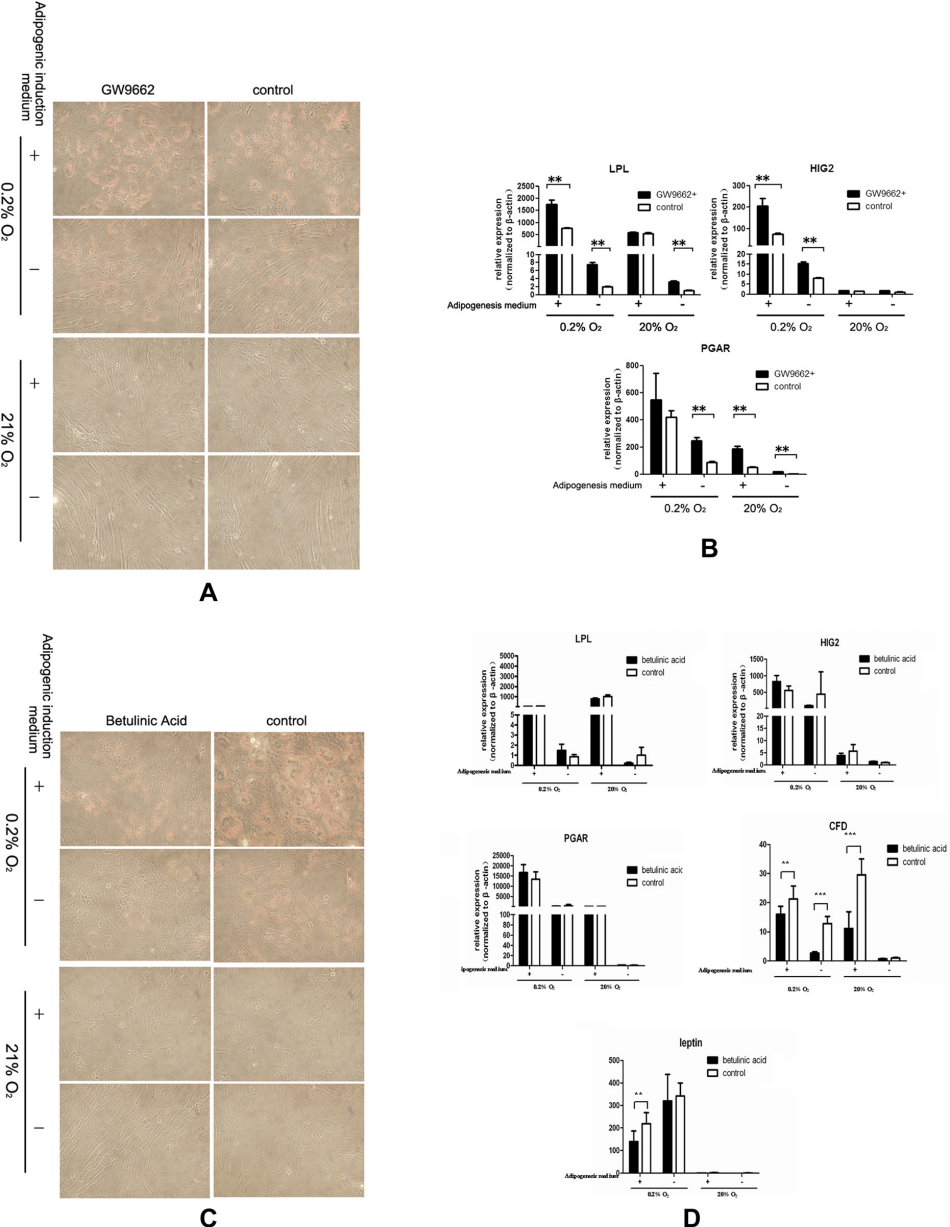
**Adipogenic differentiation of bone marrow-derived mesenchymal stem cells (BMSCs) is regulated by a C/EBP-mediated pathway and PPARγ exerts a negative effect on differentiation of BMSCs into adipocytes. (A, B)** Adipogenic differentiation of bone marrow-derived mesenchymal stem cells (BMSCs) is peroxisome proliferator activated receptor gamma (PPARγ) independent. **(A)** Oil red O staining revealed that BMSCs treated with the PPARγ inhibitor GW9662 under hypoxic conditions instead showed increased lipid droplet accumulation (magnification: 100×). **(B)** Real-time PCR assays showed that PPARγ inhibition with GW9662 induced *HIG2* and *LPL* expression. **(C, D)** CCAAT enhancer-binding proteins (C/EBPs) induced the adipogenic differentiation of BMSCs in hypoxia. **(C)** Oil red O staining revealed that BMSCs treated with the C/EBP inhibitor betulinic acid under hypoxic conditions showed less lipid droplet accumulation (magnification: 100×). **(D)** Real-time PCR assays showed that C/EBP inhibition with betulinic acid inhibited *leptin* and *CFD* expression.

As indicated by the PCR array data, under extreme hypoxic conditions BMSCs differentiate into adipocytes in a C/EBP-dependent manner, and PPARγ may inhibit this adipogenic differentiation. We then used the PPARγ inhibitor GW9662 and the C/EBP inhibitor betulinic acid to treat the cells under hypoxic conditions, and Oil red O staining and real-time RT-PCR for adipocyte-specific genes were performed. These results showed that inhibition of PPARγ using GW9662 promoted the intracellular accumulation of lipid droplets and promoted the expression of the adipocyte-specific genes *LPL* and *HIG2* (Figure 5A,B). The inhibition of C/EBPs using betulinic acid resulted in less intracellular lipid droplet accumulation (Figure 5C). Betulinic acid inhibited the expression of the adipocyte-specific genes *CFD* and *leptin* expression under hypoxic conditions, indicating that C/EBPs play crucial roles in the adipogenesis of BMSCs under extreme hypoxic conditions (Figure 5D). Loss-of-function studies showed that C/EBPs, but not PPARγ, are necessary to promote the adipogenesis of BMSCs under extreme hypoxia.

**Figure 6:**
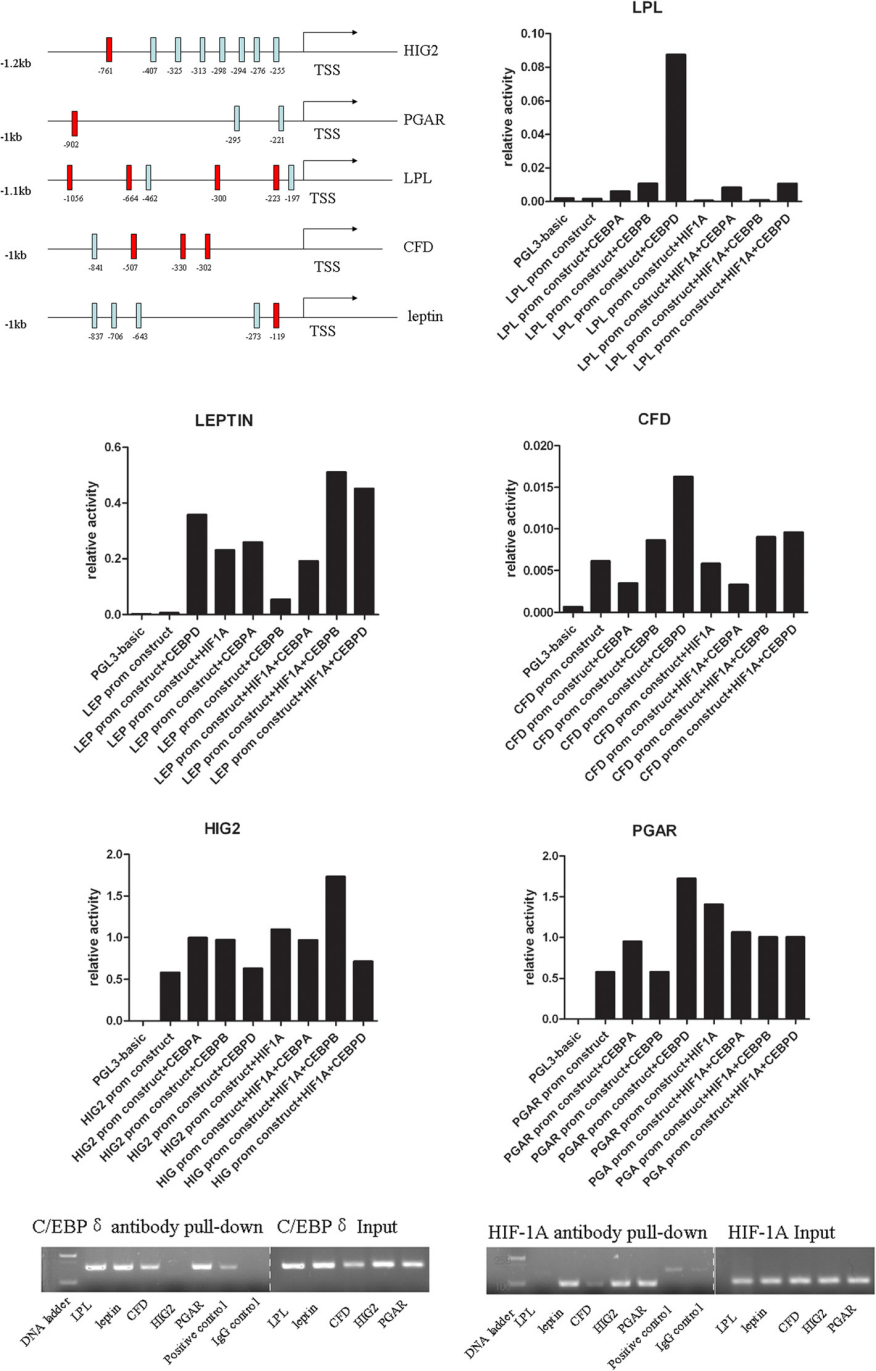
**HIF-1A and C/EBPs transcriptionally regulate the expression of adipocyte-specific genes. (A)** Predicted binding sites of hypoxia-inducible factor 1 alpha (HIF-1A; blue boxes) and CCAAT enhancer-binding proteins (C/EBPs; red boxes) in the sequence approximately 1 kb upstream of the transcription start sites (TSSs). Luciferase activity was measured 48 hours after transfection. Co-transfection with a *HIF-1A* expression vector markedly increased the reporter expression rate of *leptin*, *HIG2* and *PGAR*. Co-transfection with a C/EBPδ *expression* vector markedly increased the reporter expression rate of *leptin*, *PGAR*, *CFD* and *LPL*. HIF-1A and C/EBPβ coordinately upregulated the promoter activities of *leptin* and *HIG2*. The expression of *leptin* was also coordinately upregulated by HIF-1A and C/EBPδ. **(B)** Chromatin immunoprecipitation analysis of HIF-1A or C/EBPδ binding to the promoters was conducted. DNA released from the precipitated complexes was amplified by PCR and the PCR products were separated by agarose gel electrophoresis. Lane 1, DNA ladder; lanes 2 to 6, HIF-1A antibody or C/EBPδ antibody pull-down; lane 7, anti-RNA polymerase II; lane 8, immunoglobulin G control; lanes 9 to 13, input.

From the above-described data, we concluded that HIF-1A and C/EBPs play roles in the adipogenesis of BMSCs under hypoxic conditions. We then investigated whether HIF-1A and C/EBPs transcriptionally regulate the expression of adipocyte-specific genes, including *leptin*, *LPL*, *CFD*, *PGAR* and *HIG2*. The sequences approximately 1 kb upstream of the transcription start sites of these genes were cloned into pGL3 basic luciferase plasmids. The expected binding sites for HIF-1A and C/EBPs in the promoters of these genes were searched for using TFSEARCH [8] (Figure 6A). The clones were co-transfected with the *HIF-1A* expression vector and the *C/EBPα*, *C/EBPβ* or *C/EBPδ* expression vector. Luciferase activity was measured 48 hours after transfection. Co-transfection with the *HIF-1A* expression vector markedly increased the reporter expression rate of *leptin* (46-fold), *HIG2* (1.88-fold) and *PGAR* (2.43-fold). Co-transfection with the *C/EBPδ* expression vector markedly increased the reporter expression rate of *leptin* (71-fold), *LPL* (58-fold), *PGAR* (2.98-fold) and *CFD* (2.62-fold). HIF-1A and C/EBPβ coordinately upregulated the promoter activities of *leptin* and *HIG2*. The expression of *leptin* was also coordinately upregulated by HIF-1A and C/EBPδ. In conclusion, HIF-1A transcriptionally regulates the expression of *leptin*, *HIG2* and *PGAR*; C/EBPs, especially C/EBPδ, transcriptionally regulate the expression of *LPL*, *CFD*, *leptin* and *PGAR* (Figure 6B).

To confirm the binding of HIF-1A or C/EBPδ to the promoters of these adipocyte-specific genes, the chromatin immunoprecipitation assay was performed. A HIF-1A plasmid or a C/EBPδ plasmid was transfected into HEK293 cells. Corresponding antibodies were used to precipitate protein/chromatin complexes from sonicated samples. PCR data were obtained. As shown in Figure 6C, the binding of HIF-1A to promoter region of *leptin*, *HIG2* and *PGAR* was observed. C/EBPδ has been shown to bind to the promoter region of *LPL*, *leptin*, *CFD* and *PGAR*.

## Discussion

The presence of hypoxic regions in a solid tumor has long been known. Compared with normal tissue, oxygen in human tumors can drop to very low concentrations or even to zero. For example, the oxygen pressure level in pancreatic cancer is 2.7 mmHg (0.3%) [9]. Thus, it is reasonable to investigate tumor biology under hypoxic conditions. In cancer biology, hypoxia in the tumor microenvironment is linked to angiogenesis, proliferation, cancer stem cell niche, immune escape and metastasis. The adipogenic differentiation of BMSCs under hypoxia can be considered as a molecular adaptation to help cancer cells survive the loss of vital molecules such as oxygen or energy. The roles of hypoxia in the differentiation of MSCs remain controversial. MSCs under reduced oxygen conditions were believed to preserve their stemness and remain undifferentiated [10,11]. However, it was also reported that hypoxia enhances mesoderm lineage differentiation, including adipogenic, osteogenic or chondrogenic differentiation [12]. Hypoxia has been shown to promote or inhibit adipogenesis. MSCs showed reduced adipogenic differentiation under 1% oxygen pressure [1]. However, when MSCs were exposed to an atmosphere containing 1% of oxygen, the formation of an adipocyte-like phenotype with cytoplasmic lipid droplet accumulation was observed. However, in that case, the expression of neither the mature adipocyte-specific genes *leptin* and *adipophilin* nor the early marker gene *LPL* was induced under the hypoxic environment, indicating that despite the accumulation of the lipid droplets, true adipogenic differentiation did not occur [13]. The discrepancy may be due to the degree of oxygen deprivation and the heterogeneous nature of MSCs. In our study, to investigate the roles of BMSCs in the cancer microenvironment, we set the oxygen concentration at 0.2%, which is much lower than the oxygen concentration used in most studies. When exposed to an atmosphere containing only 0.2% oxygen, MSCs underwent obvious adipogenic differentiation, displaying not only the accumulation of lipid droplets but also the expression of the early marker gene *LPL* and the mature adipocyte-specific gene *leptin*. The expression of *LPL* decreased after long-term hypoxia treatment, indicating that LPL acts on preadipocytes during the early stage of BMSC differentiation and that hypoxia can initiate differentiation more efficiently than normoxia.

The transcription factor nuclear receptor PPARγ, the family of C/EBP and the sterol regulatory element binding protein SREBP or SREBF are believed to be crucial for conversion of precursor cells to adipocytes [14]. Several studies support the important role of PPARγ in adipocyte differentiation, identifying PPARγ as an essential and sufficient factor to induce adipocyte differentiation. However, in this study, in BMSCs under extreme hypoxic conditions of 0.2% oxygen that showed potent adipogenic differentiation, PPARγ and SREBF1 expression was obviously downregulated. The PPARγ inhibitor GW9662 enhanced the adipogenic differentiation potential of BMSCs under hypoxic conditions, indicating that the regulation of adipogenic differentiation of BMSCs under extreme hypoxic conditions is different than that under normoxia. It was reported that the expression of *PPARγ2* is repressed in a hypoxic environment [1]. The adipocyte-associated genes such as *PGAR* in white fat can be upregulated by fasting, by peroxisome proliferator-activated receptor agonists, and by hypoxia. Under extreme hypoxic conditions, rather than peroxisome proliferator activated receptor, HIF-1A and C/EBPs bind to gene promoters and increase gene expression. Hypoxia appears to exert a potent lipogenic effect that is independent of the PPARγ-regulated maturation pathway. Transcriptional responses to hypoxia are primarily mediated by hypoxia-inducible factor HIF, a heterodimer of HIF-α and the aryl hydrocarbon receptor nuclear translocator subunit [15]. Hypoxia inhibits Rb phosphorylation and blocks the DNA-binding capability of C/EBPβ to PPARγ2 in both a HIF-1A-dependent mechanism that induces p27Kip1 and a HIF-1A/p27-independent mechanism [1]. The *ob* gene product leptin is exclusively expressed in adipose tissue and is a signaling factor that regulates body weight homeostasis and energy balance [16]. Leptin can be considered a protein marker of terminally differentiated adipocytes [17]. White adipose tissue is now recognized to be a multifunctional organ; in addition to the central role of lipid storage, it has a major endocrine function, secreting several hormones, notably leptin and adiponectin, and a diverse range of other proteins. The C/EBP family is widely expressed, and its members play critical roles in the regulation energy metabolism, inflammation, hematopoiesis, cellular proliferation and differentiation. C/EBP binding sites are located within upstream regions of the human *ob* gene and drive the high-level expression of *ob* genes in adipocytes [16].

In our human adipogenesis PCR array assay, C/EBPs – especially C/EBPδ – were dramatically upregulated under the 0.2% oxygen concentration, suggesting that the C/EBP family plays roles in the adipogenic differentiation of BMSCs. The C/EBP inhibitor betulinic acid reduced lipid droplet accumulation and *leptin*, *LPL*, *CFD*, *PGAR* and *HIG2* expression under hypoxic conditions, indicating that C/EBPs transcriptionally regulate the adipogenic differentiation of BMSCs under extreme hypoxic conditions. C/EBPδ is considered an acute phase response transcription factor [18]. The expression of C/EBPδ is low or undetectable in most cell and tissue types. However, expression is rapidly induced by a variety of extracellular stimuli, such as growth hormone, insulin, interferon gamma, interleukin-1, interleukniin-6, lipopolysaccharide, TNFα and dexamethasone [19]. In the lipopolysaccharide-induced inflammatory response, C/EBPδ induced by nuclear factor-κB promotes the production of proinflammatory cytokines [20].

## Conclusions

BMSCs under extreme hypoxia show the potential to differentiate into adipocytes and high adipokine expression. HIF-1A and C/EBP, especially C/EBPδ, play important regulatory roles in the process of differentiation.

## Abbreviations

ALP: alkaline phosphatase
BMSC: bone marrow-derived mesenchymal stem cell
C/EBP: CCAAT enhancer-binding protein
HIF-1A: hypoxia-inducible factor 1 alpha
MSC: mesenchymal stem cell
PPARγ: peroxisome proliferator activated receptor gamma
